# Molecular mechanism of carbon nanotube to activate Subtilisin Carlsberg in polar and non-polar organic media

**DOI:** 10.1038/srep36838

**Published:** 2016-11-22

**Authors:** Liyun Zhang, Yuzhi Li, Yuan Yuan, Yuanyuan Jiang, Yanzhi Guo, Menglong Li, Xuemei Pu

**Affiliations:** 1 Faculty of Chemistry, Sichuan University, Chengdu 610064, People’s Republic of China; 2 College of Management, Southwest University for Nationalities, Chengdu 610041, People’s Republic of China

## Abstract

In the work, we mainly used molecular dynamics (MD) simulation and protein structure network (PSN) to study subtilisin Carlsberg (SC) immobilized onto carbon nanotube (CNT) in water, acetonitrile and heptane solvents, in order to explore activation mechanism of enzymes in non-aqueous media. The result indicates that the affinity of SC with CNT follows the decreasing order of water > acetonitrile > heptane. The overall structure of SC and the catalytic triad display strong robustness to the change of environments, responsible for the activity retaining. However, the distances between two β-strands of substrate-binding pocket are significantly expanded by the immobilization in the increasing order of water < acetonitrile < heptane, contributing to the highest substrate-binding energy in heptane media. PSN analysis further reveals that the immobilization enhances structural communication paths to the substrate-binding pocket, leading to its larger change than the free-enzymes. Interestingly, the increase in the number of the pathways upon immobilization is not dependent on the absorbed extent but the desorbed one, indicating significant role of shifting process of experimental operations in influencing the functional region. In addition, some conserved and important hot-residues in the paths are identified, providing molecular information for functional modification.

Over last three decades, biotechnological potential of nonaqueous biocatalysis has attracted considerable interests owing to its advantages like higher selectivity, thermo-stability, lower side reactions in numerous synthetic and biocatalysis^1^,^2^,^3^. Nevertheless, its applications in synthetic chemistry have been significantly limited due to low activity, recycling rate and lack of long-term operational stability in non-aqueous media. Therefore, many efforts have been devoted to develop strategies to enhance the enzyme activity, stability, and enantioselectivity. Some strategies were proposed to activate enzymes in non-aqueous media, like salt activation, chemical modification and enzyme immobilization^4^. The immobilization of enzymes is one of the most common strategies, which can enhance the catalytic properties of enzymes in both aqueous and organic media^5^,^6^,^7^. For example, α-chymotrypsin, subtilisin BPN’, and subtilisin Carlsberg immobilized on porous chitosan beads expressed higher catalytic activities than free enzymes for amino acid esterification in many hydrophilic organic solvents^8^. Subtilisin Carlsberg and α-chymotrypsin adsorbed onto silica chromatography gel support gave 1000-fold greater catalytic activities in acetonitrile media than freeze-dried powders^9^. The immobilized subtilisin Carlsberg with magnetically-separable mesoporous silica was successfully recycled for iterative synthetic model reactions in isooctane^10^.

As known, it is essential to select an appropriate carrier material in order to prepare an effective immobilized biocatalyst. The use of nanomaterials, like Gold nanoparticles (NPs), carbon nanotubes (CNTs), graphene, silica NPs, Magnetic NPs etc, as enzyme carriers is gaining a prominent place within the immobilization strategies^11^. Compared to many flat supports, CNTs can serve as excellent supporting materials for enzyme immobilization in aqueous and organic media^12^, because they offer ideal characteristics like unique electrical and mechanical properties, surface area and effective enzyme loading to improve the efficiency of biocatalysts. Consequently, the enzyme-CNT complexes display great potential applications in field of biosensors, biomedical devices and other hybrid materials^12^,^13^.

Despite of the large number of studies in this area, the mechanism at the microscopic level through which the immobilization stabilizes and enhances the activity of enzymes has not been satisfactorily elucidated on experiments owing to the complexity of the system and molecular nature of the issue. Molecular dynamics (MD) simulations can provide microscopic information regarding interactions of materials with proteins at molecular level. It has been successfully applied to study the mechanism of interaction between nanomaterials and enzymes in aqueous solution^14^,^15^,^16^. Francesco^14^ mainly used MD simulations to reveal two mechanisms of C60 blocking K^+^ channels and found that intracellular binding site with high affinity for C60 is highly conserved in K^+^ channels. Li^15^ combined experimental methods and MD simulation to study the adsorption of some serine proteases on graphene oxide (GO) and PEGylated GOs (GO-PEGs) in aqueous solution. In addition, MD simulations were also used to study interactions of single-walled CNTs with proteins^16^, amino acids^17^, kinds of peptides^18^, polysaccharides^19^, and cosolvents^20^ in aqueous solution.

As mentioned above, many researches from experiments and theories focused on the interactions between nanomaterials and biomolecules in aqueous phase, but studies on non-aqueous media have been very limited. As a result, understanding of immobilization mechanism in the organic media has been lagged and is highly desired. Subtilisin Carlsberg is a member of serine protease family and converts a large number of substrates to perform hydrolytic and transesterification reactions^10^,^21^. Also, it has been widely used for syntheses of peptides and amino acid esters in various organic solvents^21^,^22^. Thus, we, in the work, used MD simulation, binding free energy calculation and protein structure network to probe effects of CNT immobilization on the structure, substrate-binding, communication pathways for subtilisin Carlsberg (SC) in water, polar acetonitrile solvents and non-polar heptane media. Acetonitrile and heptane were chosen as representatives of polar and non-polar organic solvents in this work since they are most commonly used in non-aqueous enzyme catalysis^23^,^24^,^25^,^26^. Some observations from the work provide molecular evidences for experimental findings. More importantly, some novel observations are obtained with respect to previous studies on adsorbed enzymes^15^,^16^,^27^,^28^,^29^, which could provide valuable information for understanding the catalytic property, the specificity, the functional modification, solvent selection and some key experimental operations for the immobilized serine protease in organic media.

## Results and Discussion

In the work, three different solvent environments were considered, viz., acetonitrile (labeled as ACN), heptane media (labeled as HEP) and water solution (labeled as WAT). Six different systems were constructed. The first one is SC immobilized to one single walled carbon nanotube in water, labelled as CNT-wat. Similar to experimental operations^9^,^10^,^30^, in which the enzymes were first immobilized on carbon nanotubes in aqueous solution and then transferred to the organic solvents, the initial conformations of SC adsorbed onto CNT in the organic solvents were extracted from the final snapshot of the first 100-ns MD trajectories of CNT-wat system. Then, we transformed its solvent environment into acetonitrile (labeled as CNT-acn) and heptane (labeled as CNT-hep) media. As a reference, three free enzyme systems were also set up in aqueous (labeled as free-wat), acetonitrile (labeled as free-acn), and heptane (labeled as free-hep) media, respectively. MD simulation time is 200 ns for every system.

### Adsorption of the enzyme on Carbon Nanotubes

**Figure 1:**
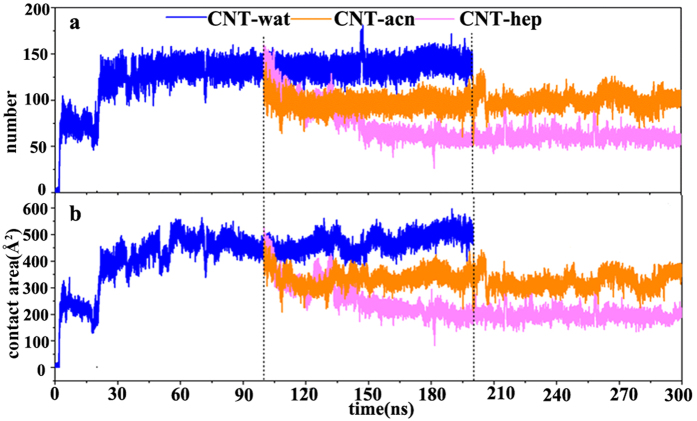
Adsorption process of subtilisin onto CNT. (**a**) The number of adsorbed atoms and (**b**) the contact area between the CNT and the enzyme with respect to the 200 ns simulation time in aqueous, acetonitrile, and heptane media for the immobilized enzymes. The initial structure of the enzyme-CNT complex in the two organic systems is derived from the final snapshot of the first 100 ns trajectories in CNT-wat system.

To gain insight into the adsorption dynamics of the subtilisin, we analyzed the time evolution of the number of adsorbed non-hydrogen atoms within 6 Å distance from the CNT surface since the distance is generally served as a criterion of hydrophobic interaction^31^. In addition, the contact area between the CNT surface and the enzyme was also calculated in terms of Eq. (1), serving as another indicator to measure the adsorption extent.


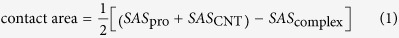


where *SAS*_pro_ and *SAS*_CNT_ are the solvent-accessible surface area (SASA) of the isolated protein and the CNT surface, respectively, and *SAS*_complex_ is that of the whole assembly of these two motifs. The result is shown in Fig. 1.

A comparison of Fig. 1(a) and Fig. 1(b) shows that the variation of the contact area is consistent with that of the number of adsorbed atoms. The adsorption of enzyme on CNT is accompanied by molecule spreading, as reflected by increasing adsorption number and contact area. It is clear that the atoms absorbed and the contact area in aqueous solution exhibit a steep increase within first 5 ns, indicating that the adsorption process is very quick. During the time period of 5–20 ns, the adsorption process reaches a relative plateau, and the number of adsorbed atoms and the contact area for the immobilized SC almost keep in ~70 and ~250 Å^2^, respectively. In the period, the immobilized enzyme should adjust its orientation or conformation for better interaction with CNT. After the temporary balance, the second jump appears between 25 ns and 27 ns due to sharply increasing number of the adsorbed atoms and the contact area. After that, the adsorption process almost achieves a relatively stable balance with ~130 adsorbed atoms and ~450 Å^2^ contact area. The multistep adsorption was also observed for three typical proteins adsorbed onto graphene^28^.

As described in Methods part, for the adsorption in the two organic media, we selected the final snapshot of the first 100-ns trajectory of the immobilized SC in aqueous solution and placed it into the acetonitrile and heptane media as an initial conformation (vide orange and pink lines in Fig. 1). Figure 1 clearly shows that the two indicators for the immobilized SC go downhill to different extents due to the change of the solvent environment. The number of adsorbed atoms and the contact area are decreased from ~130 and ~450 Å^2^ in aqueous solution to ~90 and ~300 Å^2^ in acetonitrile and ~50 and ~180 Å in heptane solvent, respectively. The extent of desorption is more significant in the hydrophobic heptane solvent, as also reflected by Supplementary Fig. S1, which displays the adsorption processes in the three solvents using some representative conformations.

**Figure 2:**
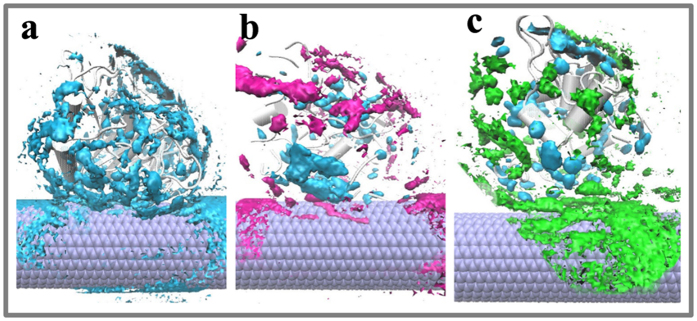
Spatial distribution of the solvent molecule around the immobilized enzyme. (**a**) Aqueous solution (**b**) Acetonitrile media (**c**) Heptane media. The enzyme corresponds to the average structure of subtilisin over the last 20 ns equilibrium trajectory for each system. Solvent color code: water (cyan), acetonitrile (rose), heptane (green).

**Figure 3:**
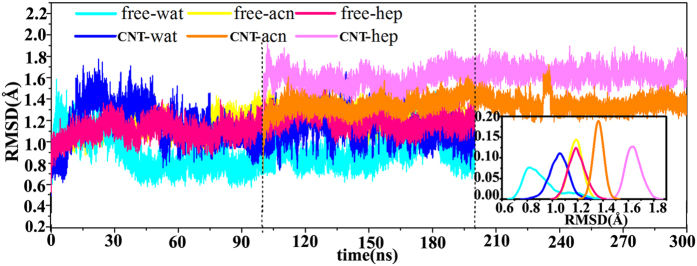
Changes in RMSD values of backbone atoms of subtilisin in the six systems. The RMSD is for deviation from the crystal structure. Inset also shows the distribution of the RMSD values.

To further investigate mechanisms of the desorption, we calculated spatial probability density distribution of water, acetonitrile and heptane molecules around the enzyme by binning atom positions from rms coordinate fit frames over all enzyme atoms at 1 ps intervals into (0.5 Å)^3^ grids over the last 20 ns trajectories in the three media, as shown in Fig. 2. It can be seen that the spatial contours enclosing high probability regions of the water molecules are mainly located at the enzyme surface and fractionally in the protein interior for the three systems. Some acetonitrile and heptane molecules diffuse into the contact region between the enzyme and the CNT, and compete with residues to interact with the CNT, which makes some residues close to the CNT in aqueous solution escape from the CNT surface, thus leading to the decrease of the adsorbed atoms and the contact areas, as revealed above. In addition, compared to the acetonitrile molecules, it can be observed from Fig. 3 that more heptane molecules spread into the contact region between the enzyme and the CNT, exhibiting stronger competition in contacting with the hydrophobic CNT surface. Some studies on adsorption of organic compounds onto CNTs in aqueous phases also reported that the non-polar solvent molecules are more likely to attach with CNTs than polar molecules due to the driving force of the hydrophobic interaction^32^,^33^,^34^, in line with our observations.

### Changes in the over all structure of SC

#### 

In order to observe the impacts of the solvent and the adsorption on the entire structure of the enzyme, RMSD values of Cα atoms for the immobilized and free SCs were evaluated, as shown in Fig. 3. It can be seen that the RMSD values of the free enzyme in the three solvents present some fluctuations in the initial several nanoseconds and then achieve a temporary constant-value after 50 ns. The average RMSD values during the last 20 ns in the two organic solvents (1.18 Å in acetonitrile and 1.20 Å in heptane) are slightly larger than that in aqueous solution (0.91 Å). On the other hand, the changes of the RMSD values of the immobilized enzyme are also consistent with the contact areas and the adsorbed atoms above. The average RMSD values of the immobilized SCs over the last 20 ns trajectories are 1.03 Å, 1.36 Å, and 1.64 Å in aqueous, acetonitrile, and heptane media, respectively, which are slightly larger than those of the free SCs in the corresponding solvents. In a whole, the changes in the entire structure of both the free and immobilized SCs are small in the three solvents, displaying strong stability of the SC structure to the environmental change, which should partly contribute to its wide applications in non-aqueous enzyme catalysis.

### Catalytic triad and substrate binding pocket

**Figure 4:**
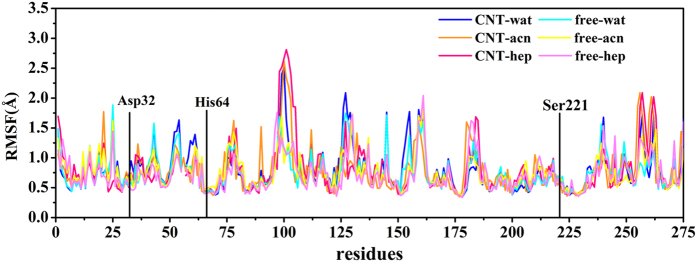


Subtilisin, as a member of serine protease family, performs its catalytic function through three catalytic triad residues (Asp32, His64, and Ser221) and substrate binding site^35^. The catalytic triad residues exhibit much lower RMSD (vide Supplementary Table S1) and RMSF values (see Fig. 4) than most residues of the SC in all systems, indicating their relative rigidities to the solvent environments and the carrier materials, which should contribute to the fact that serine protease could maintain catalytic activity in organic solvents^10^,^21^,^22^.

**Figure 5:**
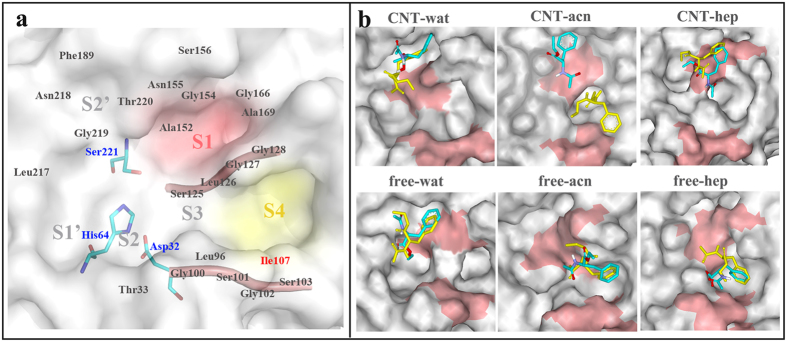
Structure of subtilisin active region and substrate binding modes after docking and MD simulations. **(a**) Schematic of subtilisin active region (including catalytic triad and substrate binding pockets). The two β-strands (Gly100-Tyr103, and Ser125-Gly128) and catalytic triad are displayed in cartoon and stick styles, respectively. **(b)** Selected binding mode of substrate based on the docking result is colored by yellow in stick style. The substrate conformation after 20-ns MD simulation is colored by element in stick style. Red and gray denote the residues of the two β-strands and the other residues around the two β-strands, respectively.

In general, the binding site of the subtilisin (S4-S2′) can be described as a surface channel or crevice that can accommodate at least six amino acid residues (P4-P2′) of a polypeptide substrate (or inhibitor)^35^. As shown in Fig. 5(a), the specificity of the substrate lines up between the extended enzyme backbone segments Gly100-Tyr103, and Ser125-Gly128, forming central strand of a three-stranded antiparallel β-sheet^36^. The S1 and S4 binding sites are two distinct and large clefts, and the substrate binding is predominantly determined by the two pockets or clefts on either side of the backbone strand Ser125-Gly128. The two sides of the S1 pocket are formed by the backbone segments consisted of Ser125-Gly128 and Ala152-Asn155, while the segment Gly166-Ala169 forms the bottom of the cleft. The S4 pocket, between the strands Ser101-Tyr104 and Leu126-Gly128, is lined with hydrophobic side chains consisted of residues Leu96, Tyr104, Ile107 and Leu126.

**Figure 6:**
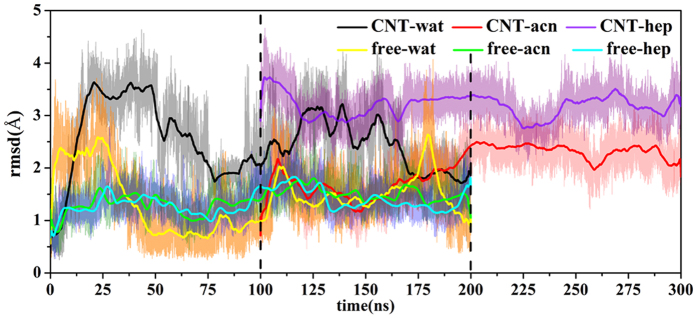
Changes in RMSD values of backbone atoms of two β-strands (Gly100-Tyr103, and Ser125-Gly128) with respect to the 200 ns simulation times for the free or the immobilized enzymes in the three different solvents. The RMSD is deviation from the crystal structure. The initial structure of the enzyme-CNT complex in the two organic systems is derived from the final snapshot of the first 100 ns trajectory in CNT-wat system.

Thus, we concerned RMSD and RMSF values of the two β-strands consisted of Gly100-Tyr103 and Ser125-Gly128 (vide Figs 4 and 6). As reflected by Fig. 6, the immobilized enzymes in the three solvents exhibit higher RMSD values than the free enzymes for the two β-strands. The average RMSD values during the last 20 ns for the immobilized enzyme are 2.1 Å in aqueous solution, 2.3 Å in acetonitrile and 3.2 Å in heptane media while these values are 1.5 Å, 1.3 Å and 1.1 Å for the free enzyme in the corresponding media, respectively, displaying the impact of the immobilization. In addition, the RMSF values of the key region Gly100-Tyr103 of the binding site are significant higher than the other residues in the six systems.

**Figure 7:**
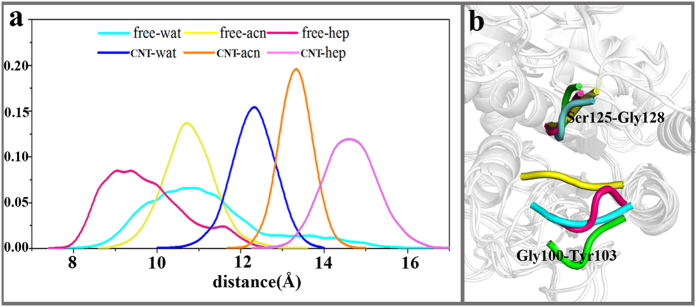
Open of the two β-strands (Gly100-Tyr103 and Ser125-Gly128) induced by the adsorption. (**a**) Distributions of the distance between the two β-strands during the last 20 ns trajectories of each simulation. **(b)** The deviation of region Gly100-Tyr103 for the immobilized enzyme. Color code: crystal structure (yellow), water (cyan), acetonitrile (rose), heptane (green).

To gain more insight into the effect of the adsorption on the conformational change of the substrate binding site, we further calculate the distance of the two β-strands, as shown in Fig. 7. The change trend in the distance distribution is also consistent with that of the aforementioned RMSD values. The distance distributions of the immobilized subtilisin concentrate in the ranges of 12 Å–13 Å in aqueous solution, 13 Å–14 Å in acetonitrile and 14 Å–15 Å in heptane, all larger than those of the free systems (10 Å–12 Å, 10 Å–11 Å, and 8.5 Å–10.5 Å) in corresponding media (vide Fig. 7(a).), indicating that the two β-strands open upon the immobilization, as further illustrated by Fig. 7(b). Peijun Ji et al^27^ used MD simulation to study lipase immobilized onto CNT in heptane media and also found that the adsorption could induces an open of lid (Thr88–Leu105) and the segment (Asn277–Leu290), favoring exposure of active site. It is also observed from Fig. 7(b) that the segment consisted of Gly100-Tyr103 is deviated from initial position to different extents, dependent on the type of solvent. The deviation is more obvious in the organic solvents, especially in nonpolar heptane. The open of the two β-strands induced by the adsorption should facilitate the enzyme to bind the substrates.

### Key residues and driving force contributed to the binding between the enzyme and CNT in the three media

To quantitatively estimate the binding strength between the subtilisin and the CNT in the three different solvents, Molecular mechanics/Poisson–Boltzmann Surface Area (MM-PBSA)^37^ analysis of the trajectories was performed to calculate the binding energy between them. The result is listed in Table 1(a). The binding free energies Δ*G*_binding_ were calculated to be −58.1 kcal mol^−1^ in aqueous, −46.3 kcal mol^−1^ in acetonitrile, and −30.7 kcal mol^−1^ in heptane media, consistent with the number of adsorbed atoms and the contact area revealed above. Analysis of binding components of the energy shows that the driving force of the binding is mainly van der Waals interactions.

**Figure 8:**
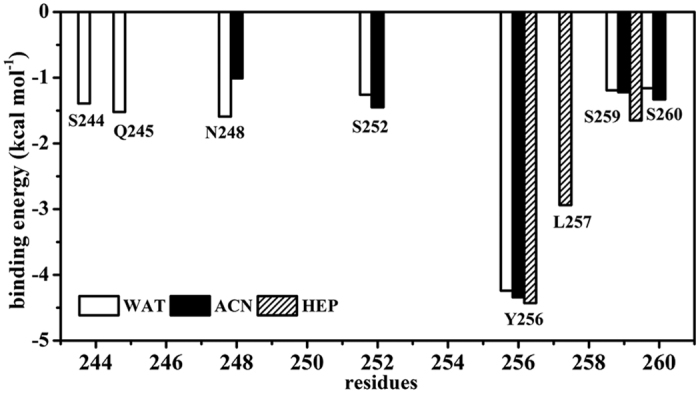
Per-residue binding energy contributed to CNT binding in aqueous (WAT), acetonitrile (ACN) and heptane (HEP) media. Only high energy residues with absolute value of binding energy above 1.0 kcal mol^−1^ are shown.

In order to identify the residues responsible for the adsorption of the subtilisin onto the CNT, we carried out a decomposition analysis of the binding energy on a per-residue basis. Figure 8 shows residues with absolute values of binding energies greater than 1 kcal mol^−1^ in the three media. As reflected by Fig. 8, the binding region of the enzyme mainly involves in Ser240–Ser265 residues, which includes members of amphipathic helix G (Ala243–Ser252) and a loop called as variable region 19 (Thr253-Gly266) in the family of subtilisin-like serine proteinases^38^. In aqueous solution, both the helix G consisted of S244, Q245, N248, and S252 and the loop region involved in Y256, S259, and S260 residues mainly contribute to the binding energy. However, when put the immobilized enzyme from the aqueous solution into the two organic media, there are significant changes in the number of favorable contribution residues and contribution extents owing to the desorption. In acetonitrile, the residues N248, S252, Y256, S259, and S260, which are observed to favor the binding in aqueous solution, still retain their favorable contribution, but with different extents. However, the contribution of the helix G residues S244 and Q245 is significantly decreased due to the slight desorption. In heptane media, the contribution of the helix G is completely disappeared due to the strong competition from the nonpolar heptane molecules with the absorbed residues in the interaction with CNT surface, while the contribution from the hydrophobic residue L257 located in the loop region is significantly enhanced.

### Effects of the immobilization on the substrate binding in different solvents

As observed above, the enzyme immobilization can lead to different extent conformational variation of the substrate binding pocket, dependent on the solvent environments. Thus, we further analyzed the effect of the conformation changes on the interaction of the SC with substrates. Docking methods were utilized to explore potential different-binding modes of subtilisin with substrates in the three different environments. We selected N-acetyl-L-phenylalanine ethyl ester (APEE) as substrate since it was revealed that the transesterification of the substrate catalyzed by subtilisin is strikingly different between nonpolar octane and polar tetrahydrofuran (THF) media^39^. For each system, 100 docking outputs were carefully analyzed (see Supplementary Fig. S2). The final docking poses are depicted in Fig. 5(b), which were obtained by considering binding energy scores and the binding modes. The result shows that the substrate mainly presents the binding with the S1 pocket of enzyme in the aqueous systems in the free-wat, CNT-wat and the CNT-hep systems, representing more than 85% population in the 100 docking results (vide Supplementary Fig. S2). And the binding modes in the free-wat, CNT-wat and CNT-hep systems also present high binding energy scores. In the binding poses, the ester group of the substrate points to one oxyanion-hole residue Asn155 and provides a hydrogen bonding interaction, which was reported to stabilize the oxyanion generated in the tetrahedral transition state^40^. Taken together, we selected the docking pose to further perform MD simulation in the three systems. Nevertheless, for the other three systems (viz., free-hep, free-acn and CNT-acn systems), the substrate is more inclined to attach to the S4 pocket with high RMSF values (vide Fig. 4), representing more than 75% population in the 100 docking result and high energy scores. Thus, we selected the complex conformation with aromatic ring of the substrate facing to the center of hydrophobic S4 pocket to further perform MD study for the free-hep, free-can and CNT-acn systems.

After the docking, 20 ns MD simulations were further performed for each complex in order to explore the dynamic behavior of the interaction between the substrate and the enzyme. It is surprising that the substrate in the immobilized enzyme system in the acetonitrile media moves from S4 site in the initial conformation to S1 site, and the other systems only slightly change their conformations, still close to the initial docking region. In other words, the binding mode of the substrate facing the S4 pocket only occurs for the free enzyme in acetonitrile and heptane solvents, further displaying the stabilization of CNT on the substrate binding.

We selected the last 5 ns trajectory to further calculate the binding free energy values between the SC and the substrate in all systems using the MM-PBSA method, which has been demonstrated to have high accuracy and good computational efficiency in calculating the binding affinities^37^. The binding free energies of the immobilized SC in water (−37.03 kcal mol^−1^), acetonitrile (−29.87 kcal mol^−1^), and heptane (−51.68 kcal mol^−1^) are higher than the corresponding free ones (−32.26, −23.96 and −29.46 kcal mol^−1^), respectively. The largest substrate-binding energy is observed in the heptane media for the immobilized SC. The increasing binding strength induced by the CNT provide further supports for the previous experiment findings that the enzyme could improve its catalytic activity by means of immobilization^7^,^8^.

**Figure 9:**
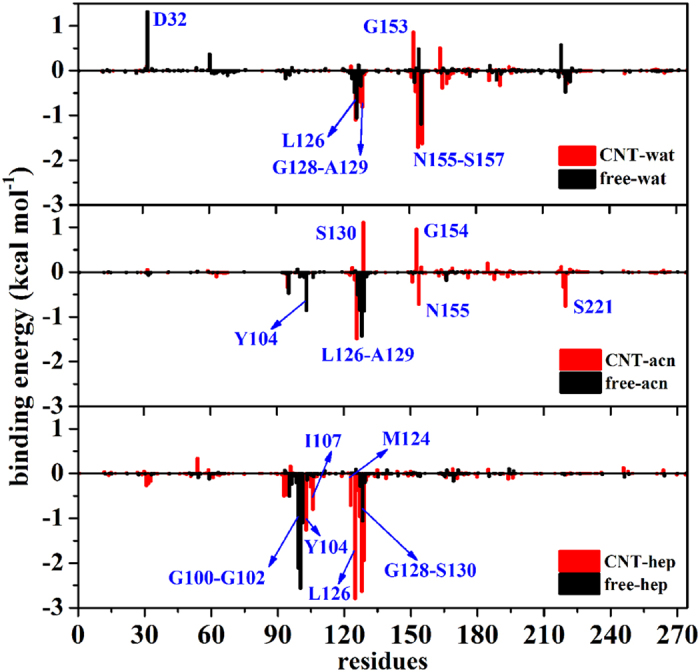
Per-residue substrate-binding energy in the six systems. Some residues significantly contributed to the binding are labelled.

**Figure 10:**
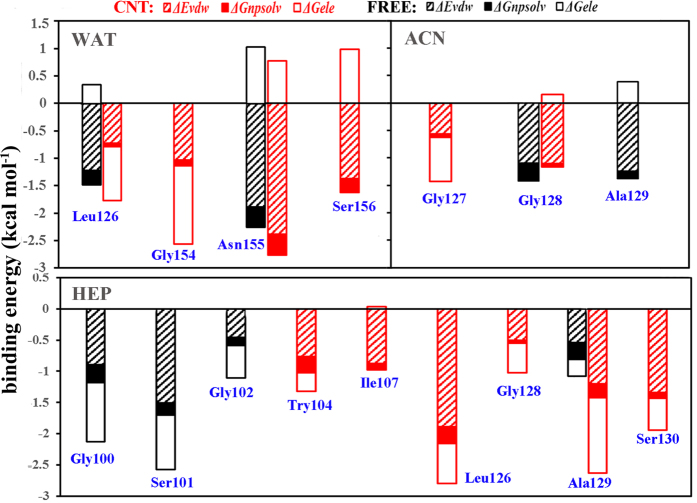
Decomposition of energy contributions into different terms for the substrate-binding. Energy decomposition into contributions from van der Waals energy (VDW), the nonpolar term of solvation energy (npsolv), and the total electrostatic energy contribution (ELE + psolv) for the residues whose absolute value of binding energy was greater than 1.0 kcal mol^−1^.

**Figure 11:**
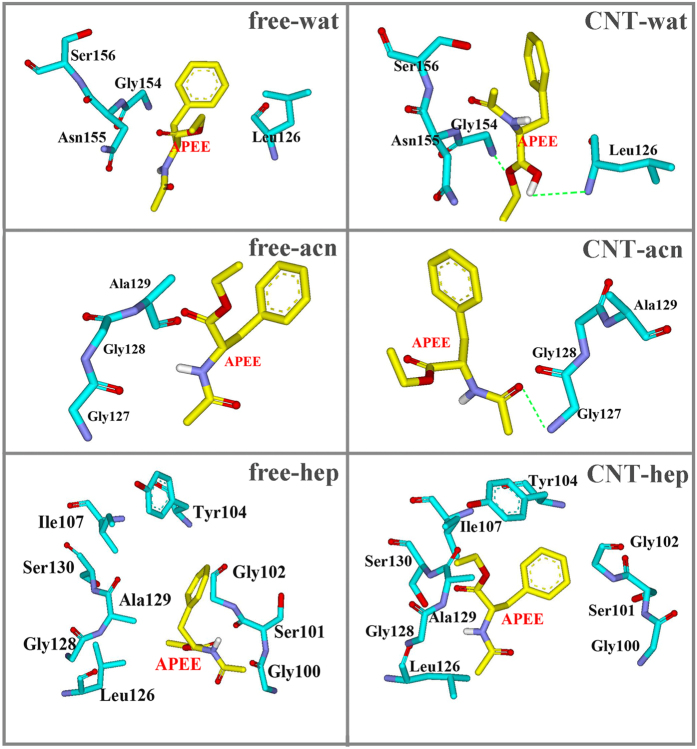


To identify important residues contributed to the substrate binding, per-residue binding energy calculations were performed on the six systems (vide Fig. 9). Figure 10 further shows the energy decompositions for the high-energy residues with the absolute values of the binding energies greater than 1.0 kcal mol^−1^. As reflected by Fig. 9, the favorable contribution region in aqueous solution mainly locates in the regions of S125–S130 and Gly154-Ser156. For the aqueous solution, it can be seen from Fig. 10 that the residues Leu126 and the oxyanion-hole residue Asn155 are observed to significantly contribute to the binding energy mainly through van der Waals interactions for the free enzyme and their favorable contributions are still preserved in the immobilized environment. Interestingly, the immobilization of CNT significantly strengthens favorable contributions from Gly154 and Ser156 residues to the substrate binding mainly through H-bonding due to the conformation changes upon the immobilization, as reflected by Fig. 10 and Fig. 11. In addition, the immobilization induces Ser156 and the substrate close, thus leading to an increase in the favorable contribution from the van der Waals interactions. As a result, the adsorption of the CNT enhances the substrate-binding strength in aqueous solution.

For the free-enzyme system in acetonitrile media, the favorable region of Gly154-Ser156 in aqueous solution is disappeared while an increase in the contribution from region Leu96-Ile107 occurs due to the conformation variation induced by the solvent environment change. When the enzyme is immobilized by the CNT in the acetonitrile media, the favorable region also mainly distributes over S125–S130 and Gly154-Ser156, similar to the aqueous solution. But, the contribution extent from the region Gly154- Ser156 is significantly smaller in the acetonitrile media than that in the aqueous solution, which should be responsible for smaller binding-energy in the acetonitrile media than the aqueous solution. A careful inspect of Fig. 10 further reveals that when put the free or the immobilized enzyme from the aqueous solution into the acetonitrile media, the type of favorable residues is entirely different from those in aqueous solution despite of some similar regions, along with significant decreases in the number of favorable residues and their contribution extents for the immobilized enzyme. As a result, the binding free energies are weakened by the acetonitrile solvent for the free and the immobilized enzymes with respect to the aqueous environment. On the other hand, for acetonitrile media, the favorable contribution of Ala129 in the free-enzyme system is disappeared. Instead, the favorable contribution from Leu127 is added mainly through the electrostatic energy, which mainly stems from the formation of H-bonding between the carbonyl oxygen of substrate and Leu127 of the enzyme (vide Fig. 11), displaying the impact of the adsorption-induced conformation changes on the substrate binding site. The contribution from the residue Gly128 to the binding is still retained. Consequently, the substrate-binding is enhanced by the adsorption of CNT in acetonitrile media, similar to the aqueous solution.

Similar to the acetonitrile media, the favorable residues also distribute over the Leu96-Ile107 or S125-S130 region in the heptane media (see Fig. 10). But, the specific residues with significant contribution in the heptane media are entirely different from the acetonitrile media. Furthermore, the number of residues of the immobilized enzyme with great contributions to the binding is obviously increased in the heptane media compared to the other two polar solvents. In addition, the total electrostatic energy contribution (Δ*G*_ele_) is favorable to the binding energy in the heptane media since the unfavorable polar contribution to the solvation free energy (Δ*G*_psolv_) is significantly weakened by the non-polar heptane molecules. As a result, the immobilized enzyme in the heptane media exhibits the highest substrate-binding energy among all the six systems, which should favor its catalytic activity. Also, it may be assumed that the activity of the immobilized enzyme is possibly higher in the non-polar solvent than the polar organic solvent, similar to the observation from its free type and some other free enzymes^39^. As revealed by Fig. 10, the residues significantly contributed to the binding in the free enzyme system are quite different from the immobilized one in heptane media due to the impact of the CNT adsorption. The β-strand residues Gly100-Try103 and residue Ala129 significantly contribute to the binding energy for the free enzyme. Whereas for the immobilized enzyme, only the contribution of Ala129 is reserved and enhanced while the contribution from the region Gly100-Try103 is disappeared due to the departure of the β-strand residues Gly100-Try103 from their initial positions (vide Fig. 11), causing the substrate to interact with partial residues of S1 and S4 binding sites alternatively. Compared to the free enzyme system, the immobilization leads to more residues favorably contributed to the substrate binding like the hydrophobic residues Try104, Ile107, Leu126 and some nearby residues Gly128 and Ser130, leading to larger binding energy.

### Communication Pathways to the active region derived from PSN analysis

In order to probe the mechanism regarding the role of the adsorption in influencing the active region (viz., the binding pocket and the catalytic triad residues), we utilized protein structure network (PSN) method^41^ to study structural communication paths to the active region in the three different media. PSN as a graph-based approach was proposed and defined by Vishveshwara and co-workers^41^. The method can provide network features (e.g., nodes, hubs, and links) and gain insight into the global properties of protein dynamics, topological rearrangements and functionally important residues. Consequently, it has been widely applied to study protein folding, protein stability, internal communications, allosterism and so on^42^,^43^,^44^.

The communication pathways between the active region and the other residues, which are served as the two extremities to search paths, were obtained by DCCM analysis based on the last 20-ns trajectory for each system (see Method part for details). Wordom algorithm was used to determine the shortest path between selected pairs of vertices in a graph. Table 2 lists dominant optimal pathways that accounts for more than 30% frequency in the total communication pathways. For the substrate-binding region, we mainly focused on the two β-sheets due to their significant variations observed above.

It can be seen that the total number of pathways in the two organic media is significantly greater than those in the aqueous solution, either for the free enzyme or the immobilized one, indicating that the communication paths to the active region are strengthened after shifting the aqueous environment to the two organic media. In addition, compared to the free enzyme system, the adsorption of CNT increases the number of the communication pathways to the active region in the three media, which should be responsible for larger variation in the substrate-binding pocket upon immobilization, as revealed above. Similar to the total pathways, the number of pathways to the two β-strands and catalytic triad are increased by the immobilization. Especially, the paths to G100-Y103 are significantly enhanced and the increase extent is significantly larger than that of S125-G128 (vide Table 2), which should contribute to the significant deviation of G100-Y103 from the initial position upon the immobilization, as observed above. However, the immobilization only slightly increases the number of the paths to the catalytic triad in the three solvents, displaying minor impact. As a consequence, the catalytic triad retains the relative rigidity in the immobilization.

**Figure 12:**
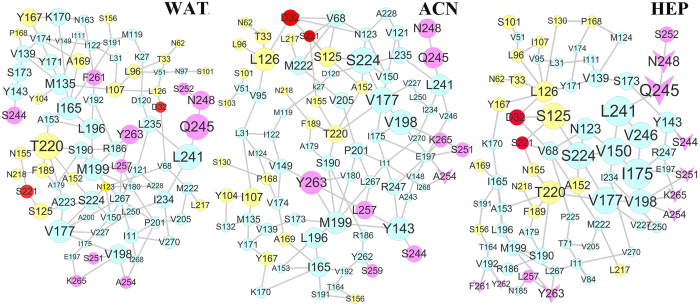
Protein structure networks (PSN) for the immobilized subtilisins in the three solvents. Community networks from the adsorbed residues of SC (rose node) in the final snapshot of the 100 ns trajectories of CNT-wat system to the catalytic triad (red node) and the substrates binding pocket residues (yellow node) in aqueous (WAT), acetonitrile (ACN), and heptane (HEP) media, derived from the last 20-ns trajectories. The other residues in the pathways were colored as cyan. The residues desorbed in the organic media are displayed as V-shaped nodes. The size of the node is in proportion to the frequency of appearance in the pathways.

Compared the immobilized systems in the three media, it is confused that the conformation changes of the substrate binding pocket induced by the immobilization is the most significant in the heptane media, but the adsorbed atoms are least in the media. We have to meet the question: why? In other words, how does the immobilization influence the substrate-binding pocket? In order to probe the origin, we paid attention to the shift process of the immobilized enzyme from the aqueous solution to the organic media since the significant desorption was observed above, in particular for the non-polar heptane solvent. We conjectured that the conformation variation induced by the desorption should be one main reason. In order to confirm the assumption, we further analyzed the communication pathways from the adsorbed residues in the aqueous solution to the substrate-binding site in the three media. The adsorbed residues within 6 Å distance around the CNT surface at the final snapshot of the first 100 ns trajectory of CNT-wat system, which was served as the initial conformation for the simulation in the organic solvents, was selected as starting nodes and the substrate-binding residues act as ending nodes. The paths were searched between the starting nodes and the ending ones in the three media for the immobilized systems, based on the last 20 ns trajectories of 200 ns simulations. The PSN-path graphic results are shown in Fig. 12, in which the key residues identified by PSN for in the adsorbed region of the final snapshot of the first 100 ns trajectories of CNT-wat system, catalytic residues and the substrate-binding residues were colored as rose, red and yellow, respectively, and the others were colored as cyan. The V-shaped nodes denote desorbed residues in the organic media (See Supplementary Table S2 for details). The size of the node is in proportion to the frequency of appearance in the pathways.

As reflected by Fig. 12, the absorbed residues N248, Q245, and Y263 involved in a large amount of communication pathways to the active region in aqueous solution, in which T220 in the substrate-binding site is most frequently visited. In the acetonitrile media, only one crucial desorption residue A254 is retained in the main pathway but not presenting significant impact, as judged from its small size of node in Fig. 12. The important impact of absorbed residues N248, Q245 and Y263 identified in aqueous solution still remains in the acetonitrile media. The attendance frequency of T220 is decreased by the acetonitrile media while the substrate-binding residues S125 and L126 located in S1 and S4 binding site show higher visited-frequency. In addition, the attendance frequencies of hydrophobic residues Y104 and I107 located at S4 binding site are to some extent enhanced. Consequently, there is a larger separation between the two β-strands (S125-G128, G100-Y103) in the acetonitrile than the aqueous solution, as revealed above. Several experimental studies^45^,^46^ confirmed that mutations at Y104, I107, and L126 could modulate the substrate preference. Therefore, it can be assumed that the specificity of the immobilized subtilisin should be also influenced to some extent by the polar organic solvent, similar to the free enzyme. However, after shifting the immobilized enzyme from the aqueous solution to the non-polar heptane media, only two adsorbed residues S252 and L257 are retained in the main pathways due to stronger desorption. Inversely, the nine desorbed residues (vide Fig. 12) play a more important role in influencing the active region since 83% of the total communication pathways start from the nine desorbed residues, in particular for the desorbed residues N248 and Q245. Similar to the acetonitrile system, the visited frequencies of substrate binding site residues S125, L126 and S101 are significantly enhanced and higher than those in the acetonitrile media. Consequently, the largest expansion between the two β-strands is observed for the immobilized SC in the heptane media.

On the other hand, the main communication pathways from the adsorbed region in aqueous solution to the catalytic triad show that the residues D32 and S221 present low attendance-frequency in aqueous solution, as reflected by their small node-sizes in Fig. 12. The visited-frequency of D32 in the two organic solvents is to different extent increased with larger extent in the heptane media, main through enhancing the link to L126. But, the weight of the catalytic residue D32 in the main paths is much smaller than many other crucial residues, as judged from its small node-size in Fig. 12, thus also contributing to the low RMSD and RMSF values of the catalytic site. In addition, although there are significant differences in the pathways among the three media, some common subnets are still shared by the immobilized enzyme despite of the different media like N248 = >Q245 = >L241 and V198 = >V177 = >S224, as reflected by Fig. 12, indicating their conserved and important roles in the paths to the active region. Thus, it can be assumed that mutations on these residues will significantly modulate the bioactivity and specificity for the immobilized subtilisin.

In a whole, PNS analysis reveals that the significant expansion between the two β-sheets induced by the immobilization of CNT is dependent on the desorbed extent in the shifting process from the aqueous solution to the organic media, rather than the adsorbed extent.

## Conclusion

In this work, we mainly used molecular dynamic simulations to study the subtilisin Carlsberg (SC) immobilized onto CNT in aqueous, acetonitrile, and heptane media in order to probe effects of the adsorption on the enzyme structural and functional variations, in turn providing useful information for understanding the activation mechanism of the enzyme upon immobilization. As a reference, the free SC in the three types of media was also simulated in the work.

The result indicates that SC can be strongly absorbed onto CNT within dozens nanoseconds in aqueous solvent. When shifted to the organic solvents, desorption quickly occur to different extents, in particular for the non-polar heptane solvent, as confirmed by less adsorption atoms, smaller contact area and lower binding free energies. The driving force of the adsorption of SC onto CNT is mainly van der Waals interactions. Some organic molecules could diffuse into the contact region between the enzyme and CNT, and compete with residues to interact with the hydrophobic CNT surface, in particular for the non-polar heptane molecules with stronger competition ability than the polar acetonitrile ones. As a result, larger desorption and weaker binding with CNT are observed in the non-polar heptane solvent compared to the polar media. The catalytic triad residues of SC display to be insensitive to the immobilization of CNT and the solvent environment since the change in the outside environment plays minor role in influencing the communication pathways from the other regions to them, which should be responsible for the fact that the enzymes still reserve their catalytic activity in non-aqueous environments. In contrast, the immobilization plays a significant role in influencing the structure of the substrate binding site. The distances between the two β-strands (Gly100-Tyr103, and Ser125-Gly128) in the substrate binding site are expanded upon the immobilization in the three solvents, which should facilitate the entrance of substrate, in turn favoring the substrate-binding. The increase extent of the distance is most significant in the heptane media compared to the polar water and acetonitrile ones. Indeed, the immobilized SCs exhibit higher affinities with the substrate compared to the free enzymes in all the three media. Much higher binding energy is observed for the immobilized SC in the non-polar heptane media than the other two polar solvents, resulted from more residues contributed to the substrate-binding and a significant decrease in the unfavorable polar contribution due to the non-polar solvent environment. Compared to the aqueous solution, the polar acetonitrile solvent decreases the number of favorable residues contributed to the substrate binding, leading to lower substrate-binding energy either for the free or the immobilized SC. The protein structure network (PSN) analysis further reveals that the increase in the distance between the two β-strands is attributed to the fact that the immobilization enhances the structural communication paths to the binding pocket, thus inducing larger separation. More importantly, our PSN analysis further reveals that the impact of the immobilization on the active region in the organic solvents is not dependent on the adsorbed extent but the desorbed one, which are closely associated with the shifting process in experimental operations. Consequently, the distance between the two β-strands is the largest in the heptane media due to the most significant desorption in the media. In addition, the PSN analysis also identified some important and conserved hot-residues and links in these main paths to the active region, like N248 = >Q245 = >L241 and V198 = >V177 = >S224, implying their importance in modulating the bioactivity for the immobilized subtilisin. The novel observations from this work will provide valuable information for exploring activation mechanism of immobilized enzyme and advancing activation and functional-modification strategies in field of non-aqueous enzymatic catalysis.

## Method

All MD simulations were carried out using the AMBER 12 package^47^. The ff99SB force field^48^ was used for subtilisin Carlsberg (SC).

### Construction of initial models

The crystal structure of the subtilisin Carlsberg was obtained from Protein Data Bank (PDB ID 3UNX^49^) at 1.26 Å resolution, which contains 274 residues and 265 crystal water molecules. All water molecules in the crystal structure were retained. The titratable protein side chains were assigned to be their standard protonation state using H + + 3.0^50^ Web Server (http://biophysics.cs.vt.edu/) at pH 8.0, consistent with the experimental conditions^10^. The single walled carbon nanotube with chirality (12, 12) was built using VMD package^51^, in which C-C bond length is defined as 1.42 Å and pipe diameter size is about 1.6 nm. We choose one single walled CNT with length of about 8.0 nm enough to adsorb the subtilisin, which contains1584 carbon atoms. The carbon atoms of the CNT were modeled as uncharged lennard-jones particles^52^ using sp^2^ carbon parameters of the ff99SB force field. The initial coordinates of acetonitrile and heptane were optimized with Gaussian 09^53^ program at the HF/6–31G* level, and the restrained electrostatic potential (RESP) charge fitting procedure was used to derive atomic charges. General Amber force field (GAFF) was assigned for the optimized structures.

Initially, the minimum distance between the subtilisin Carlsberg and the CNT surface was more than 12 Å. Experimental studies^54^ indicated that the enzyme immobilized with the active site oriented toward solution showed a higher catalytic efficiency compared to random oriented immobilization. Thus, we placed the active site side of the enzyme facing opposite to the CNT. Extra water and organic solvent molecules were added using LEAP utility. The enzyme or CNT-enzyme was solvated in a rectangle periodic box of water, acetonitrile or hexane with 12 Å, 15 Å, and 22 Å, respectively. As a result, the number of water, acetonitrile and heptane molecules in CNT-complex systems are 17658 for CNT-WAT, 5148 for CNT-ACN, 2289 for CNT-HEP and the corresponding values for the free systems are 11781, 3670 and 1633 in water, acetonitrile and heptane media, respectively. Counter ions Na^+^ were added to neutralize the whole systems.

### Molecular Dynamics Simulations

To remove bad contacts in the initial geometry of each system, three energy minimizations (5000 steps for the solvent molecules followed by 5000 steps for the protein/CNT, finally, 5000 steps for the whole system) were done by the same procedure: the steepest descent method for the first 3000 steps for relaxation of the close contacts, followed by conjugate gradient algorithm for the remaining part. After minimizations, the systems were heated from 0 to 300 K within 120 ps using Berendsen temperature coupling^55^ with the backbone atoms of enzyme and CNT restrained by a 10 kcal mol^−1^ Å^−2^ position harmonic potential. Then, the system was equilibrated for 5 ns at 300 K in NVT ensemble without any restriction. Finally, 195 ns subsequent MD simulations were carried out in isothermal isobaric (NPT) ensemble, keeping the temperature at 300 K and constant pressure (1 atm). The integration step for MD simulations was set to 2 fs. Particle mesh Ewald (PME)^56^ method was applied to treat electrostatic interactions with a 12 Å non-bonded cutoff. All bond lengths involving in hydrogen atoms were constrained by using SHAKE algorithm^57^ with a tolerance of 1.0× 10^−5 ^Å. The atomic coordinates were saved every 2 ps for further analysis.

For the six systems under study, the energy minimizations and the MD simulations were performed with the same steps and parameters above. All of the trajectories were analyzed using analysis module of AMBER 12.0 and some other developed MD analysis programs.

### Molecular Docking Calculations

In order to study the impact of CNT on the interaction between the substrate and the enzyme in the three solvents, substrate (N-acetyl-L-phenylalanine ethyl ester) was docked into the free enzyme and the enzyme-CNT complex using Autodock^58^ program. The substrate was considered to be flexible. The pdbqt files of enzyme and substrate were prepared in AutoDockTools from the above-mentioned complexes. We selected reasonable conformations from molecular docking optimization models with binding energy scores in the top ten. Then 20 ns NPT MD-simulations were performed for the docking conformations in order to perform the free energy calculation.

### MM–PB(GB)SA Calculations

MM-PBSA method calculates the interaction energy between two molecules with molecular mechanics and estimates the solvation free energy. The binding free energy ΔG_binding_ was estimated using Eq. (2)


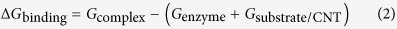


where *G*_complex_, *G*_enzyme_ and *G*_substrate/CNT_ are the free energies of the complex, enzyme, and substrate (CNT), respectively. The free energies are determined with SANDER program from Amber 12 and represent sum of the following terms (see Eq. 3)


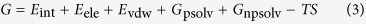


The first three terms express the internal, electrostatic and van der Waals interaction energies, respectively, sum of which represent gas-phase energy. *G*_psolv_ denotes the polar contribution to the solvation free energy, which was calculated by solving Poisson-Boltzmann equation. *G*_npsolv_ represents the nonpolar solvation energy, which was calculated as γ × *SASA*, where γ is the surface tension parameter (γ = 0.0072 kcal Å^−2^) and *SASA* is the solvent-accessible surface area of the molecule. *T* denotes the absolute temperature and *S* is the molecule entropy. Similar to many computational investigations^59^,^60^,^61^, the entropy term was not included in our free energy calculations since we more concerned with the relative change of the binding affinity.

The solute interior dielectric constant was set to be 1 and the exterior solvent dielectric constant values were set to be 80 for water, 37.5 for acetonitrile and 1.92 for heptane solvents. The average binding energy was calculated in terms of 500 snapshots extracted every 10 ps from the last 5-ns MD trajectories.

Per-residue decomposition analysis was performed by MM–GBSA module in the AMBER 12. It is a non-perturbing alternative method, which estimates the contribution of each residue to the binding energy by means of component analysis. The contributions of molecular mechanical energies and solvation free energies are assigned to atoms participated to the respective interaction. Then, summation over atoms of each residue discloses its contribution to the binding free energy.

### Dynamical cross-correlation matrices (DCCM)

Correlation plots were obtained by computing Cα dynamical cross-correlation matrix (DCCM). *C*(*i, j*) was calculated from the last 20 ns trajectory of the 200 ns simulation for each system using the following equation:


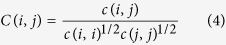


where *C*(*i,j*) is the covariance matrix element of the protein fluctuation between residues i and j. We obtained an average *C*(*i, j*) matrix based on cutoffs of 0.4 (in absolute values) to evaluate the pair of both positively and negatively correlated residues characterized by the most relevant averaged correlation values. To carefully verify that the analysis of an average *C*(*i, j*) matrix did not cause a loss of relevant information, the consistency between the average *C*(*i, j*) matrix and the individual matrices used in the averaging was evaluated.

### Protein Structure Network (PSN)

The PSN method^41^ employs graph formalism to define a network of interacting residues in a given protein or protein complex. In the method, interaction strength *I*_*ij*_ between non-covalently interacting atoms are used as edge weights, where *i* and *j* are residue identifiers. This value is calculated on the basis of the number of distinct atom pairs between residues *i* and *j* within a distance cutoff of 5.0 Å:


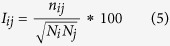


Where *n*_*ij*_ is number of distinct atom pairs between the side chains of amino acid residues *i* and *j. N*_*i*_ and *N*_*j*_ are normalization values for residues *i* and *j* obtained from a statistically significant protein dataset. PSN network was calculated for each frame from the last 20-ns MD trajectory, in which the shortest path between selected pairs of nodes was analyzed. The shortest path was identified as the path, in which the two residues were non-covalently connected by the smallest number of intermediate nodes. Only the shortest paths, in which at least one node identified possesses a significant correlation value (0.4) with one of the residues of the selected pair, were retained. All the PSN and PSN-DCCM calculations were performed using WORDOM^62^ MD trajectories analysis suite.

## Additional Information

**How to cite this article**: Zhang, L. *et al*. Molecular mechanism of carbon nanotube to activate Subtilisin Carlsberg in polar and non-polar organic media. *Sci. Rep.* **6**, 36838; doi: 10.1038/srep36838 (2016).

**Publisher's note**: Springer Nature remains neutral with regard to jurisdictional claims in published maps and institutional affiliations.

## Supplementary Material

Supplementary Information

## Figures and Tables

**Table 1:** The component and standard errors of binding energy (in kcal mol^−1^) between (a) CNT and subtilisin, (b) substrate and subtilisin in water, acetonitrile and heptane media.

Contribution	(a) CNT-subtilisin			(b) Substrate-subtilisin					
	CNT-wat	CNT-acn	CNT- hep	CNT-wat	Free-wat	CNT-acn	Free-acn	CNT-hep	Free-hep
Δ*E*_ele_^a^	0.0 ± 0.0	0.0 ± 0.0	0.0 ± 0.0	−12.0 ± 1.1	−12.6 ± 0.9	−16.9 ± 1.2	−8.8 ± 0.6	−12.5 ± 1.2	−12.1 ± 1.9
Δ*E*_vdw_^b^	−87.2 ± 2.9	−67.2 ± 3.4	−33.2 ± 3.9	−26.2 ± 2.1	−21.4 ± 1.6	−18.0 ± 1.1	−13.5 ± 1.8	−25.1 ± 2.6	−10.2 ± 1.6
Δ*E*_int_^c^	−0.0 ± 0.0	−0.0 ± 0.0	−0.0 ± 0.0	−0.0 ± 0.0	−0.0 ± 0.0	−0.0 ± 0.0	−0.0 ± 0.0	−0.0 ± 0.0	−0.0 ± 0.0
Δ*E*_gas_^d^	−87.2 ± 2.9	−67.2 ± 3.4	−33.2 ± 3.9	−38.2 ± 3.9	−34.0 ± 2.9	−34.9 ± 3.1	−22.3 ± 2.5	−37.2 ± 3.6	−22.3 ± 2.3
Δ*G*_npsolv_^e^	−7.6 ± 0.3	−5.9 ± 0.2	−2.7 ± 0.2	−21.6 ± 1.8	−20.9 ± 0.7	−17.3 ± 1.0	−14.8 ± 0.7	−22.7 ± 1.6	−13.1 ± 1.2
Δ*G*_psolv_^f^	36.7 ± 1.7	26.9 ± 1.4	5.3 ± 0.6	22.8 ± 0.9	22.6 ± 1.1	20.3 ± 1.3	15.1 ± 0.7	8.2 ± 0.6	6.0 ± 0.7
Δ*G*_solv_^g^	29.1 ± 1.6	20.9 ± 1.2	2.5 ± 0.4	1.2 ± 0.1	1.8 ± 0.2	7.0 ± 0.8	1.7±0.2	−14.5 ± 0.7	−7.2 ± 0.8
Δ*G*_ele_^h^	36.7 ± 1.7	26.8 ± 1.4	5.3 ± 0.6	10.7 ± 0.3	10.0 ± 0.5	7.5 ± 0.5	6.4 ± 0.3	−3.8 ± 0.3	−6.1 ± 0.2
Δ*G*_binding_^i^	−58.1 ± 2.5	−46.3 ± 2.8	−30.7 ± 3.6	−37.0 ± 3.6	−32.3 ± 2.9	−31.9 ± 3.2	−26.0 ± 2.3	−51.7 ± 3.8	−29.5 ± 2.3

^a^Non-bonded electrostatic energy as calculated by the MM force field.

^b^Non-bonded van der walls contribution from MM force field.

^c^Internal energy arising from bond, angle, and dihedral terms in the MM force field.

^d^Total gas phase energy.

^e^Nonpolar contribution to the solvation free energy.

^f^Polar contribution to the solvation free energy calculated.

^g^Solvation free energy;

^h^Total electrostatic energy contribution to the binding energy.

^I^Binding energy Δ*E*_gas_ = Δ*E*_ele_ + Δ*E*_vdw_ + Δ*E*_int_, Δ*G*_solv_ = Δ*G*_npsolv_ + Δ*G*_psolv_ Δ*G*_ele_ = Δ*E*_ele_ + Δ*G*_psolv_, Δ*G*_binding_ = Δ*E*_gas_ + Δ*G*_solv._

**Table 2:** The number of communication pathways between the active region and the other residues more than the frequency of 30%.

	Cnt-wat	Free-wat	Cnt-acn	Free-acn	Cnt-hep	Free-hep
Total^a^	2263	2101	3301	3030	2985	2814
G100-Y103^b^	50	0	162	33	130	0
S125-G128^c^	194	155	295	271	298	260
catalytic triad^d^	123	107	190	188	175	165

^a^The number of pathways from the other residues to the active region consisted of the substrate-binding pocket and the three catalytic residues.

^b^The number of pathways from the other residues to G100-Y103.

^c^The number of pathways from the other residues to S125-G128.

^d^The number of pathways from the other residues to the three catalytic residues.
