# Supplementary material for: Molecular mechanism of carbon nanotube to activate Subtilisin Carlsberg in polar and non-polar organic media
Source: Sci Rep. 2016 Nov 22;6:36838. doi: 10.1038/srep36838 (PMC5118797; doi:10.1038/srep36838)
Supplement: Supplementary Information [file srep36838-s1.pdf]

Supplementary Information

Molecular mechanism of carbon nanotube to activate Subtilisin Carlsberg in polar and non-polar organic media

Liyun Zhang [a], Yuzhi Li [a], Yuan Yuan [b], Yuanyuan Jiang [a], Yanzhi Guo [a], Menglong Li [a], Xuemei Pu [a]*

[a]Faculty of Chemistry, Sichuan University, Chengdu 610064, People's Republic of China

[b] College of Management, Southwest University for Nationalities, Chengdu 610041, People's Republic of China.

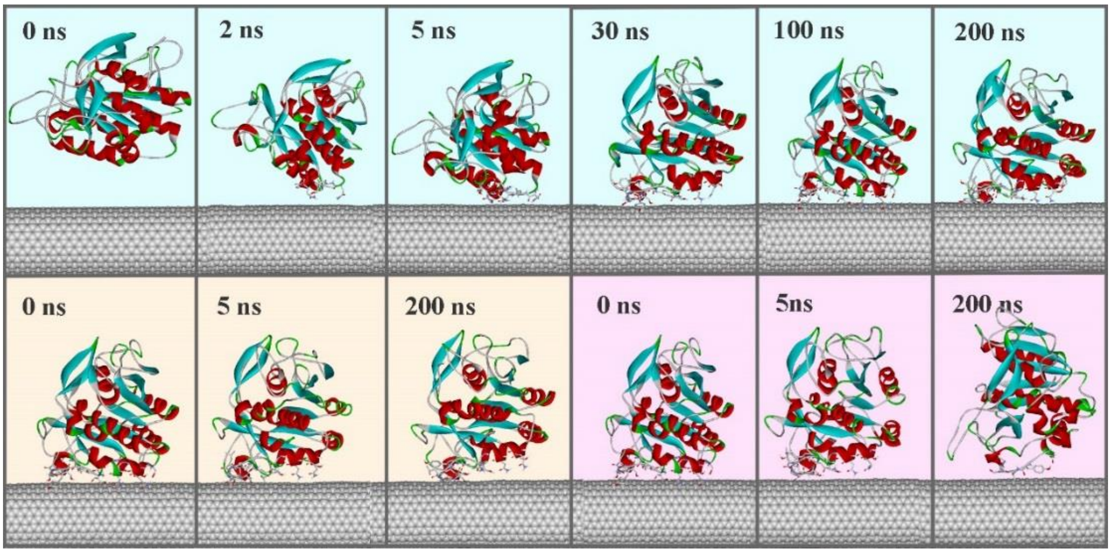

Supplementary Figure S1. The representative snapshots selected from the 200 ns simulation trajectories on basis of the extent of adsorption and desorption of the immobilized subtilisin in aqueous (top), acetonitrile (left bottom), and heptane (right bottom) media. Subtilisin is displayed in ribbon style and colored by secondary structure type. The CNT is displayed in CPK style. The residues within 6 Å around the CNT surface are displayed in line style. The initial structure of the enzyme-CNT complex in the two organic systems is derived from the snapshot of 100th ns trajectories in CNT-wat system.

Supplementary Table S1. The average RMSD values of backbone atoms of the catalytic triad (viz., Asp32, His64 and Ser221) during the last 20 ns trajectories in aqueous, acetonitrile, and heptane media for the free and immobilized enzymes. The RMSD is for deviation from the crystal structure.

	CNT-wat	free-wat	CNT-acn	free-acn	CNT-hep	free-hep
RMSD (Å)	0.51	0.23	0.36	0.38	0.53	0.44

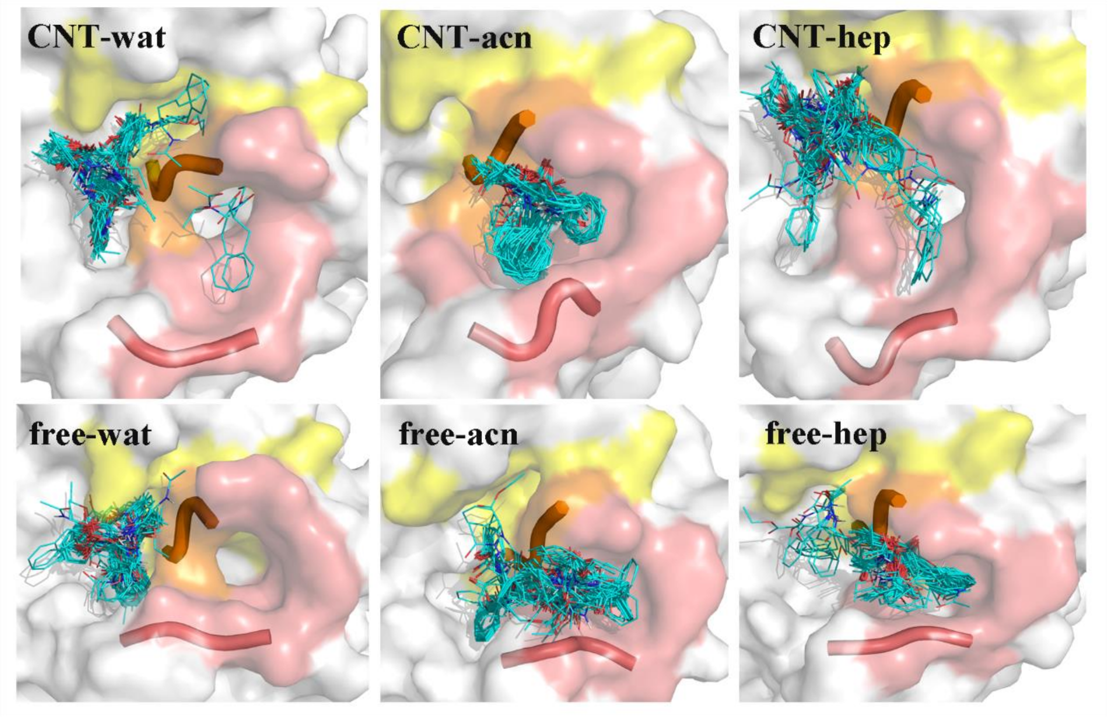

Supplementary Figure S2. 100 substrate-docking poses for the six systems. The two β-strands (S125-G128, G100-Y103) are displayed in cartoon style. Yellow and pink denote the residues of the S1 and S4 binding site, and orange denotes the common residues of the two binding site.

Supplementary Table S2. Details for the absorbed residues by CNT and the desorbed residues in aqueous, acetonitrile and heptane media.

	CNT-wat	CNT-acn	CNT-hep
Absorbed residues	Tyr143, Ser242, Ser244, Gln245, Asn248, Ser251, Ser252, Ala254, Tyr256-Phe261, Tyr263-Lys265	Ser244, Gln245, Asn248, Ser251, Ser252, Tyr256-Phe261, Tyr263-Lys265	Ser252, Tyr256-Ser260,
Desorbed residues		Tyr143, Ser242, Ala254	Tyr143, Ser242, Ser244, Gln245, Asn248, Ser251, Ala254, Phe261, Tyr263-Lys265
